# Reduced Secretion of YopJ by *Yersinia* Limits In Vivo Cell Death but Enhances Bacterial Virulence

**DOI:** 10.1371/journal.ppat.1000067

**Published:** 2008-05-16

**Authors:** Igor E. Brodsky, Ruslan Medzhitov

**Affiliations:** Howard Hughes Medical Institute and Section of Immunobiology, Yale University School of Medicine, New Haven, Connecticut, United States of America; Tufts University School of Medicine, United States of America

## Abstract

Numerous microbial pathogens modulate or interfere with cell death pathways in cultured cells. However, the precise role of host cell death during in vivo infection remains poorly understood. Macrophages infected by pathogenic species of *Yersinia* typically undergo an apoptotic cell death. This is due to the activity of a Type III secreted effector protein, designated YopJ in *Y. pseudotuberculosis* and *Y. pestis*, and YopP in the closely related *Y. enterocolitica*. It has recently been reported that *Y. enterocolitica* YopP shows intrinsically greater capacity for being secreted than *Y. pestis* YopJ, and that this correlates with enhanced cytotoxicity observed for high virulence serotypes of *Y. enterocolitica*. The enzymatic activity and secretory capacity of YopP from different *Y. enterocolitica* serotypes have been shown to be variable. However, the underlying basis for differential secretion of YopJ/YopP, and whether reduced secretion of YopJ by *Y. pestis* plays a role in pathogenesis during in vivo infection, is not currently known. It has also been reported that similar to macrophages, *Y. enterocolitica* infection of dendritic cells leads to YopP-dependent cell death. We demonstrate here that in contrast to *Y. enterocolitica*, *Y. pseudotuberculosis* infection of bone marrow–derived dendritic cells does not lead to increased cell death. However, death of *Y. pseudotuberculosis*–infected dendritic cells is enhanced by ectopic expression of YopP in place of YopJ. We further show that polymorphisms at the N-terminus of the YopP/YopJ proteins are responsible for their differential secretion, translocation, and consequent cytotoxicity. Mutation of two amino acids in YopJ markedly enhanced both translocation and cytotoxicity. Surprisingly, expression of YopP or a hypersecreted mutant of YopJ in *Y. pseudotuberculosis* resulted in its attenuation in oral mouse infection. Complete absence of YopJ also resulted in attenuation of virulence, in accordance with previous observations. These findings suggest that control of cytotoxicity is an important virulence property for *Y. pseudotuberculosis*, and that intermediate levels of YopJ-mediated cytotoxicity are necessary for maximal systemic virulence of this bacterial pathogen.

## Author Summary

The ability of bacterial pathogens to modulate death of infected host cells is an important virulence determinant. For pathogenic members of the genus *Yersinia*, the type III secreted effector protein YopJ/YopP is required for *Yersinia*-induced macrophage death. The YopJ protein is expressed by *Y. pseudotuberculosis*, while the ninety-four percent identical YopP protein is expressed by *Y. enterocolitica*. *Y. enterocolitica* infection also triggers YopP-dependent killing of dendritic cells, which are critical antigen presenting cells of the immune system. We demonstrate that in contrast to macrophages, dendritic cells are resistant to *Y. pseudotuberculosis*-mediated cytotoxicity. However, *Y. pseudotuberculosis* expressing YopP in place of YopJ was highly cytotoxic toward dendritic cells. This difference in cytotoxicity was attributable to a difference in the delivery of YopJ and YopP into mammalian cells. Furthermore, mutation of two amino acids at the N-terminus of YopJ enhanced its delivery and cytotoxicity. Remarkably, we found that enhancing the cytotoxicity of *Y. pseudotuberculosis* by expression of YopP led to its attenuation in a mouse model of *Yersinia* infection. This indicates that optimal virulence for a given pathogen requires careful regulation of virulence properties and highlights the potential evolutionary tradeoffs between cellular cytotoxicity and in vivo virulence.

## Introduction

It is generally thought that the ability of bacterial pathogens to cause host cell death in vitro is important for virulence in vivo [1]. However the role of host cell killing in bacterial virulence during in vivo infections remains poorly understood. Whether a particular optimal level of cell death might exist during bacterial infection, and how such a level might be achieved is a question that has not yet been addressed. Standard genetic approaches to investigate the basis of microbial pathogenesis typically screen or select for loss of function mutants in which a particular virulence property is completely abrogated. However, modulating the level of activity of individual virulence factors by bacterial pathogens has been shown to be important for maximizing overall virulence. This may be because of two opposing selective pressures that act on any given virulence property: positive selection for enhanced bacterial replication is countered by a selection for reduced host detection. Thus, because host organisms possess mechanisms to sense infection and are likely to respond to disruption of signaling pathways, bacterial pathogens must evolve additional mechanisms for modulating an initial baseline activity of particular virulence factor. For example, during the process of cell invasion, the activity of the *Salmonella* GEF protein SopE is counteracted by the phosphatase SptP [2], and this activity of SptP is important for limiting TNF-α production by *Salmonella* infected cells [3]. Additionally, replacement of the *Listeria monocytogenes* pore-forming toxin LLO with the related but much more active toxin PFO leads to attenuated bacterial replication in vitro and in vivo [4]. We wished to examine whether bacterial proteins that trigger cell death might also possess an optimal level of activity for a given bacterial pathogen. We therefore devised a system in which the cytotoxicity of the facultative extracellular pathogen *Yersinia pseudotuberculosis* could be altered in a tunable fashion. This approach enabled us to uncover a requirement for limiting the extent of cell death during the course of animal infection that has not previously been appreciated in studies of *Yersinia* virulence.

The Gram negative genus *Yersinia* contains three pathogenic species whose close evolutionary relationship provides an ideal system with which to investigate the evolution of bacterial virulence [5]. *Y. pestis* (the etiologic agent of plague) and *Y. pseudotuberculosis* are the most closely related, with *Y. pestis* believed to have recently evolved from *Y. pseudotuberculosis* [5]. *Y. enterocolitica* comprises a more distantly related, heterogeneous group of biotypes that are both highly pathogenic and non-pathogenic in animal infection models. A common feature of *Yersinia* infection is a tropism for lymphoid tissues. Interestingly, despite the shared enteric lifestyle of *Y. pseudotuberculosis* and *Y. enterocolitica*, highly virulent serotypes of the latter appear to trigger more acute pathology in infected tissues[6], and have a 10-50-fold lower LD_50_ in experimental mouse infections [7],[8]. This difference in pathology and virulence characteristics may reflect diverse selection pressures faced by these two species over the course of their evolution away from a common ancestor.

All three pathogenic *Yersinia* species harbor a virulence plasmid of approximately 70 kB that encodes a Type III Secretion System (TTSS) as well as secreted effector proteins designated *Yersinia* outer proteins or Yops [9],[10]. The virulence plasmid interferes with key immune functions during infection [11],[12],[13],[14]. However, the tropism of *Yersinia* species for lymphoid tissue appears to be dependent upon bacterial chromosomal factors, as bacteria lacking the virulence plasmid can still colonize mesenteric lymph nodes following oral infection [15].

Among the best studied aspects of *Yersinia* infection is its cytotoxicity toward mammalian macrophages. This cytotoxicity is caused by a type III secreted effector protein, designated YopJ in *Y. pestis* and *pseudotuberculosis* and YopP in *Y. enterocolitica* [16],[17]. YopJ and YopP interfere with NF-kB and MAPK signaling pathways in infected cells, leading to a block in cytokine secretion and macrophage death [13],[18]. Studies have suggested that the mechanism of YopJ activity lies in its ability to deubiquitinate signaling proteins, including TRAF6 and IKKβ, that are required for NF-κB and MAPK responses to bacterial infection 19,20. More recent studies propose that YopJ/P acylates substrates such as MKK6 and MEK2 which prevents their subsequent phosphorylation and activation [21],[22]. Regardless of the precise mechanism, it is clear that one of the major consequences of *Yersinia* infection is the YopJ/P-dependent death of infected macrophages, and that YopJ contributes to systemic virulence following mouse oral infection [23].

Despite the close evolutionary relationship between the pathogenic *Yersiniae*, heterogeneity exists among *Yersinia enterocolitica* isolates with respect to the presence or absence of a high-affinity iron-transport system encoded by the high pathogenicity island (HPI) [24],[25], and presence or absence of YopT among *Y. pseudotuberculosis* isolates [26]. It has also been observed that YopP of different *Y. enterocolitica* serotypes can differ in enzymatic activity due to the presence of an arginine/serine polymorphism at amino acid 143 [27]. The presence of R143 correlates with high virulence among *Y. enterocolitica* isolates [28]. Although YopJ of *Y. pestis* and *Y. pseudotuberculosis* contains the R143 polymorphism associated with higher activity, it was recently shown that YopP from the high-virulence *Y. enterocolitica* O∶8 serotype is more cytotoxic to macrophages than YopJ of *Y. pestis*, and expression of YopP in *Y. pestis* increases its in vitro cytotoxicity [29]. This difference in cytotoxicity was shown to be due to an increase in the secretion of YopP relative to YopJ of *Y. pestis*. However, the basis for reduced secretion of YopJ, and whether control of Yop secretion plays a role in *Yersinia* virulence in vivo remains unknown.

Studies on the interaction of *Y. enterocolitica* and DCs have suggested that like macrophages, DCs are highly susceptible to killing by *Yersinia* infection, and that this killing is dependent upon the activity of YopP [30]. Other studies have indicated that killing of DCs by *Y. enterocolitica* interferes with the priming of adaptive T cell responses in infected mice [31]. More recent work has further suggested that blocking of MAPK pathways by YopP in DCs infected by *Y. enterocolitica* prevents DC pinocytosis and antigen uptake [32].

Our initial examination of *Y. pseudotuberculosis* infection of DCs did not show high levels of cell death, although in accordance with previous studies, macrophages showed significant levels of cell death in response to the same infectious dose of *Y. pseudotuberculosis*. Our studies therefore reveal that DCs are markedly more resistant than macrophages to induction of cell death by *Y. pseudotuberculosis*, in contrast to previous reports indicating that both DC and macrophage cytotoxity is limited during *Y. pestis* infections [29],[33]. We suspected that *Y. pseudotuberculosis* may thus have evolved a species-specific means of reducing its cytotoxicity toward DCs. We demonstrate that two amino acid polymorphisms in the N-terminus of YopJ and YopP are responsible for controlling their levels of secretion and translocation, which correlates directly with the extent of cytotoxicity of *Y. pseudotuberculosis* relative to *Y. enterocolitica* and their differential ability to inhibit MAPK activation in infected dendritic cells. The biological importance of this reduced DC cytotoxicity was revealed upon infection of mice with *Y. pseudotuberculosis* strains that differed in their cytotoxicity toward DCs: surprisingly, we found that enhancing cytotoxicity of *Y. pseudotuberculosis* resulted in its attenuation following oral infection. This level of attenuation was similar to that seen with a *yopJ* deficient strain of *Y. pseudotuberculosis* [23]. These data suggest that maximal virulence of *Y. pseudotuberculosis* requires an intermediate level of YopJ-mediated cytotoxicity.

## Results

### Dendritic cells are more resistant than macrophages to apoptosis induced by YopJ but not YopP

**Figure 1:**
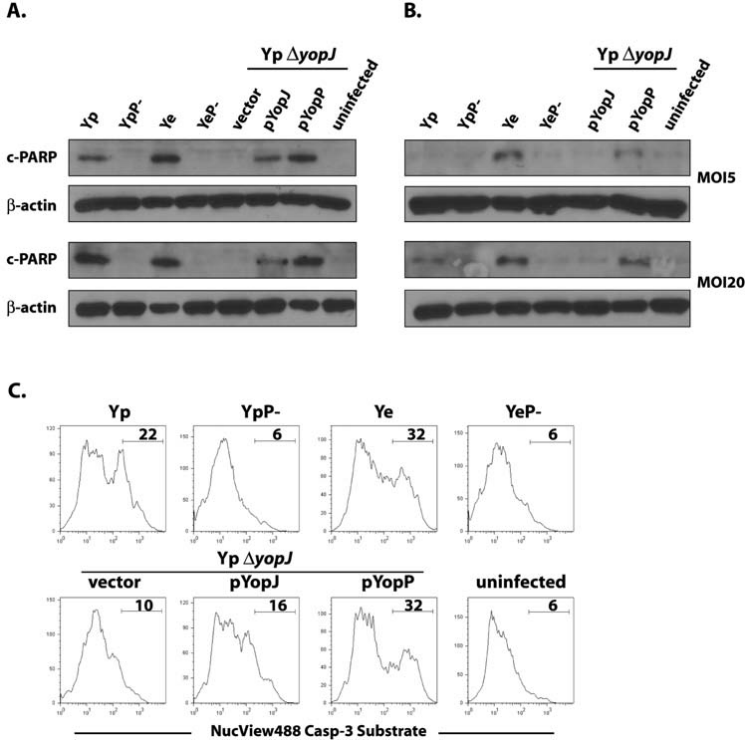
*Y. enterocolitica* YopP induces apoptosis more potently than *Y. pseudotuberculosis* YopJ in dendritic cells. (A) PARP cleavage was measured by immunoblotting of macrophage and (B) dendritic cell lysates after infection with indicated *Y. pseudotuberculosis* (Yp) and *Y. enterocolitica* (Ye) strains at indicated multiplicity of infection (MOI). P- indicates bacteria lacking the virulence plasmid. Immunoblots are representative of 3 independently infected preparations of cells. (C) NucView™ 488 Caspase-3 fluorescent substrate labeling of dendritic cells infected at an MOI of 5 with indicated bacterial strains.

We initiated our studies by examining the extent of cell death in macrophages and dendritic cells infected with *Y. pseudotuberculosis* and *Y. enterocolitica*. Macrophage death following infection with *Yersinia* occurs through caspase-3 by a pathway involving Bid cleavage [34]. We therefore examined cleavage of the Caspase-3 substrate poly-ADP ribose polymerase (PARP) as a means of comparing the extent of cell death in macrophages and dendritic cells (DCs). PARP was cleaved in macrophages infected either with *Y. enterocolitica* or *Y. pseudotuberculosis* at low and high MOI, as expected (Figure 1A). However, DCs infected with *Y. pseudotuberculosis* showed no cleavage of PARP at low MOI, and only very low levels of PARP cleavage at high MOI. This was in contrast to DCs infected with *Y. enterocolitica*, which had high levels of PARP cleavage at both low and high MOI (Figure 1B). Death of *Yersinia*-infected macrophages is mediated by the YopJ/YopP proteins [16],[17]. We therefore used the low copy plasmid pACYC184 to complement a *yopJ* mutant of the *Y. pseudotuberculosis* strain IP2666 with either *yopJ* (called pYopJ) from *Y. pseudotuberculosis* or *yopP* (called pYopP) from *Y. enterocolitica*. Complementation with pYopJ or pYopP resulted in PARP cleavage in *Y. pseudotuberculosis* infected macrophages; however, only expression of YopP in *Y. pseudotuberculosis* enabled PARP cleavage in infected DCs (Figure 1A and 1B). We confirmed that caspase-3 was indeed differentially activated in DCs infected by these various strains of *Yersinia* using a cell-permeable fluorescent caspase-3 substrate. Consistent with the extent of PARP cleavage, a much greater percentage of cells activated caspase-3 following infection with either *Y. enterocolitica* or *Y. pseudotuberculosis* expressing YopP than cells infected with *Y. pseudotuberculosis* expressing YopJ (Figure 1C). Macrophages were not more permissive for Yop translocation than DCs, as equivalent levels of a YopE-β lactamase reporter fusion protein were translocated into both macrophages and DCs (Table S2). It is also unlikely that differential attachment of *Y. pseudotuberculosis* to DCs accounts for the observed difference, as expression of YopP alone in *Y. pseudotuberculosis* was sufficient to cause markedly increased caspase-3 activation and PARP cleavage in DCs.

### MAPK activation in dendritic cells is more potently inhibited by YopP than YopJ

The observation of increased PARP cleavage and caspase-3 activation in DCs infected with *Y. pseudotuberculosis* expressing YopP but not YopJ suggested an intrinsic difference between YopJ and YopP. Recent studies showed that although *Y. pestis* and *Y. enterocolitica* infection show equivalent inhibition of macrophage p38 MAPK activation, *Y. pestis* YopJ and *Y. enterocolitica* YopP are differentially translocated into infected macrophages [29]. Macrophages infected by *Yersinia* are thought to undergo cell death due to inhibition of NF-κB and MAPKs by YopJ/P in the context of stimulation by bacterial LPS [35],[36]. This is due to the requirement for NF-κB and MAPK signaling in the synthesis of anti-apoptotic gene products following treatment with pro-inflammatory stimuli [37].We therefore examined MAPK activation in dendritic cells following *Yersinia* infection.

**Figure 2:**
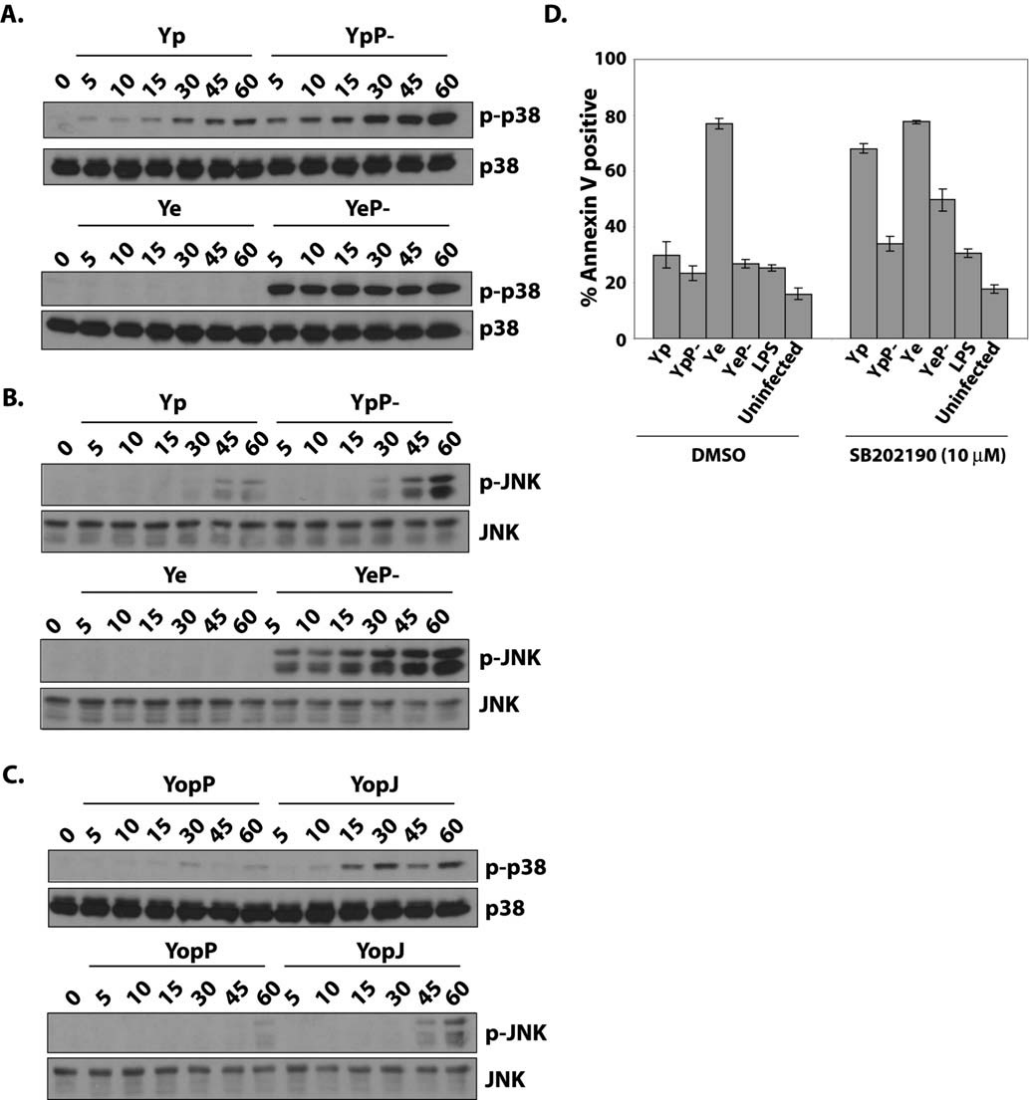
YopP inhibits MAPK activation to a greater extent than YopJ. (A) Cell lysates from dendritic cells infected for indicated number of minutes with wild type *Y. pseudotuberculosis* or *enterocolitica* (Yp or Ye, respectively) or their virulence plasmid-deficient counterparts (YpP- or YeP-) were probed for phospho-p38 and total p38. (B) Identical cell lysates as in (A) were probed for phospho-SAPK/JNK and total SAPK/JNK. (C) Cell lysates from dendritic cells infected with Yp Δ*yopJ* expressing either YopP or YopJ as indicated were probed for phospho- and total-MAPK proteins as in (A) and (B). All immunoblots are representative of three independently performed experiments. (D) Flow cytometry analysis of annexinV-positive DCs treated with vehicle (DMSO) or the p38 inhibitor SB202190 immediately prior to being left untreated, treated with LPS, or infected with Yp, YpP-, Ye, or YeP-. Data are represented as bar graphs of the mean of triplicate samples, and are representative of three independently performed experiments.

As anticipated, DCs infected with virulence plasmid-deficient bacteria of either species rapidly phosphorylated p38 and SAPK/JNK; in contrast, wild-type *Y. enterocolitica* completely blocked p38 and SAPK/JNK phosphorylation but *Y. pseudotuberculosis* did not (Figure 2A and B). This suggested that in contrast to prior observations in macrophages, dendritic cells are differentially sensitive to MAPK inhibition by YopJ or YopP. Indeed, the increased inhibition of MAPK activation in DCs infected with *Y. enterocolitica* was specifically due to YopP, as expression of YopP alone in *Y. pseudotuberculosis* was sufficient to inhibit p38 and SAPK/JNK phosphorylation to virtually the same extent as *Y. enterocolitica* (Figure 2C). Notably, kinetics of MAPK activation in cells infected with virulence plasmid- deficient bacteria differed markedly between *Y. enterocolitica* and *Y. pseudotuberculosis*: activation of both p38 and SAPK/JNK was much more rapid in YeP- infected cells than YpP- infected cells. This may be due to subtle differences in the LPS of *Y. enterocolitica* and *Y. pseudotuberculosis*, as heat killed bacteria showed similar differences in kinetics of MAPK activation (data not shown). We did not observe differential inhibition of NF-κB activation in DCs infected with *Y. enterocolitica* and *Y. pseudotuberculosis* (data not shown). This suggested that increased MAPK inhibition was primarily responsible for the increased death of DCs infected by *Y. enterocolitica*. Indeed, specific inhibition of p38 in the context of wild-type *Y. pseudotuberculosis* infection was sufficient to increase dendritic cell death to nearly the same extent as cells infected by *Y. enterocolitica* (Figure 2D). That this effect was not due to nonspecific increase in death was demonstrated by LPS-treated DCs or DCs infected with *Y. pseudotuberculosis* lacking the virulence plasmid, which did not show dramatically increased death upon p38 MAPK inhibition. Together with the PARP cleavage, these data indicated that an intrinsic difference between the YopJ and YopP proteins was most likely responsible for the differences in phenotypes observed between *Y. enterocolitica* and *Y. pseudotuberculosis*.

### Enhanced dendritic cell death in *Y. enterocolitica*-infected cells is due to N-terminal polymorphisms that enhance secretion and translocation of YopP relative to YopJ

**Figure 3:**
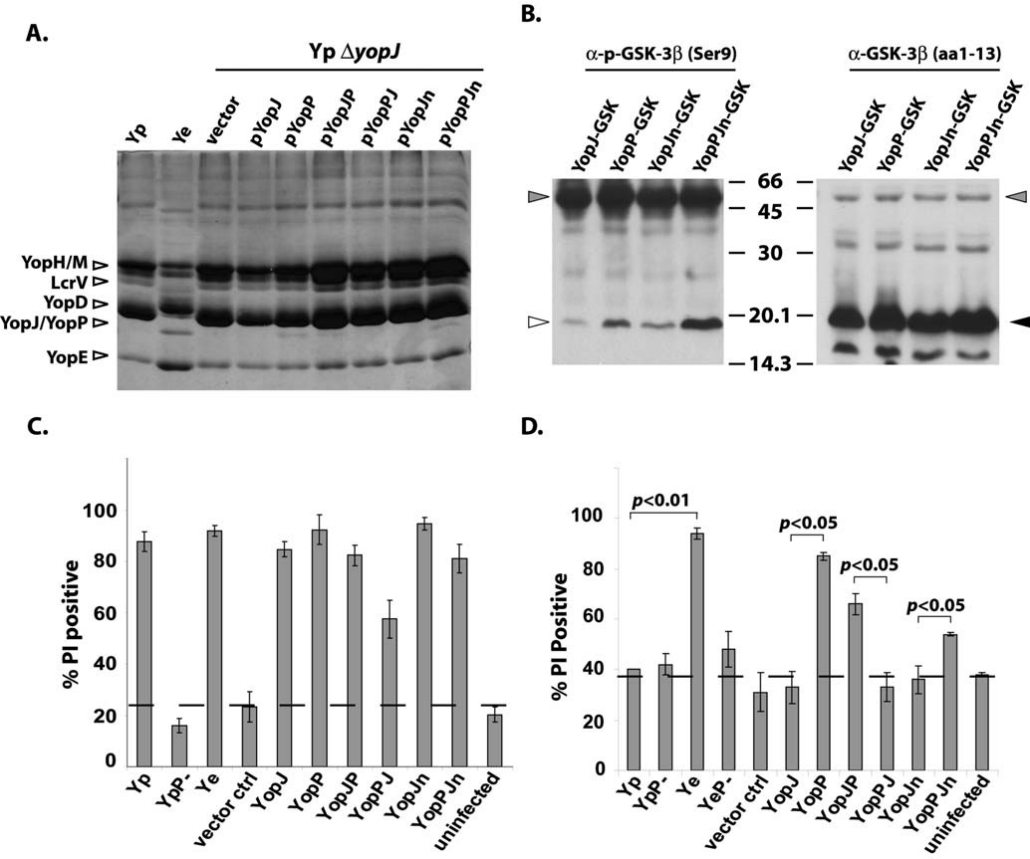
Extent of YopJ/YopP secretion correlates with degree of dendritic cell death following *Yersinia* infection. (A) TCA-precipitated supernatants from indicated bacterial strains analyzed by SDS-PAGE and Coomassie blue staining. (B) Cell lysates from HeLa cells infected with *yopJ* mutant *Y. pseudotuberculosis* expressing indicated GSK fusion proteins analyzed by Western blotting for levels of translocation of GSK fusion protein (anti-phospho-Ser9-GSK) and for levels of total GSK fusion protein (anti-GSK3β aa1-13). White arrow indicates phospho-GSK fusion protein, black arrow indicates total GSK-tagged fusion protein. Grey arrows indicate endogenous phospho-GSK and GSK. (C) Percentage of bone marrow derived macrophages that stain positively with annexinV/propidium iodide (PI) 8 hrs post-infection at MOI of 20 with indicated bacterial strains. Dotted line indicates background staining of uninfected cells. (D) Percentage of bone marrow derived dendritic cells that stain positively with PI 16–18 hrs post-infection at MOI of 20 with indicated bacterial strains. Graphs are representative of 3 independently infected preparations of cells, performed in triplicate. *p* values obtained by Student's unpaired two-tailed t-test.

Recent work has indicated that *Y. pestis* YopJ and *Y. enterocolitica* YopP are differentially translocated into infected macrophages when expressed from a strong promoter on a high-copy plasmid [29]. However, the basis for this differential translocation is unknown. We therefore examined the in vitro secretion of Yops from a *yopJ* mutant of *Y. pseudotuberculosis* expressing YopJ or YopP on a low copy plasmid, as well as from wild-type *Y. pseudotuberculosis* and *Y. enterocolitica*. Similarly to published observations [38],[39], YopJ of *Y. pseudotuberculosis* was poorly detected in bacterial culture supernatants, in contrast to YopP which was easily observed by SDS-PAGE of TCA-precipitated *Yersinia* culture supernatants (Figure 3A). YopP ectopically expressed in *Y. pseudotuberculosis* also was secreted at higher levels than YopJ (Figure 3A). The *yopP* coding sequence alone was sufficient for this enhanced secretion, since replacement of the *yopJ* protein coding sequence with that of *yopP*, generating a plasmid designated pYopJP, resulted in markedly greater levels of protein secretion than the pYopJ or the converse pYopPJ plasmid (Figure 3A). A schematic diagram of the construction of pYopJP and pYopPJ is provided in supporting information (Figure S1A).

Type III secretion in *Yersinia* is thought to depend upon both mRNA and primary amino acid sequences within the first 10–15 codons/amino acids of the secreted effector protein [40],[41],[42],[43]. We examined this region of YopP and YopJ for insight into the basis of their differential secretion. The coding sequences of *yopP* and *yopJ* have four nucleotide polymorphisms within this region (Figure S1B). Two of these polymorphisms are synonomous changes, while two are non-synonomous, resulting in an IS to SP change at amino acids 10 and 11. We therefore generated mutations in the *yopJ* coding sequence of plasmids pYopJ and pYopPJ, creating substitutions in all 4 polymorphic nucleotides. These plasmids were designated pYopJn or pYopPJn, and demonstrate that whereas wild-type YopJ is poorly secreted, mutation of 4 nucleotides at the 5′ end of *yopJ* is sufficient to markedly enhance secretion of YopJ (Figure 3A, compare YopPJ to YopPJn). We separated the non-synonomous and synonomous mutations by making constructs pYopPJ2aa, with IS→SP amino acid changes in YopJ, and pYopPJ2nt, with two nucleotide changes in *yopJ* but no alteration of the YopJ protein sequence (Figure S1C). Mutation of IS→SP was in fact sufficient to enhance YopJ secretion, indicating that the YopJ/P amino acid sequence was primarily responsible for the level of secretion (Figure S1D). In order to measure the amount of protein actually translocated into infected cells, we constructed reporter plasmids encoding a GSK tag in the pYopJ, pYopP, pYopJn and pYopPJn plasmids. This tag is phosphorylated by host cell Ser/Thr kinases only upon translocation of the fusion protein into the cytosol, and is therefore a useful tool to measure the extent of protein translocation [44]. Indeed, we observed that the translocation of YopP and YopPJn was significantly higher than that of YopJ and YopJn, as detected by levels of phospho-GSK fusion protein (Figure 3D, left panel). Furthermore, levels of translocated YopJn were notably higher than levels of translocated YopJ. The amount of total GSK fusion protein was similar for all of the constructs (Figure 3D, right panel), indicating that differences in steady-state level of total protein were not responsible for differences in translocation.

We next examined the extent of cell death caused by infection with *Y. pseudotuberculosis* strains harboring these plasmids. As expected and consistent with the known role of of YopJ/P in macrophage death, an increase in macrophage cell death occurred upon infection with any of the strains containing pYopJ, pYopP or chimeric and mutant, but not vector control, plasmids, demonstrating their functionality (Figure 3C). In contrast, death of DCs occurred only when they were infected by strains containing plasmids mediating high levels of YopJ/P protein secretion, specifically pYopP, pYopJP, and pYopPJn (Figure 3D). These plasmids also mediate high levels of Yop/P protein translocation, suggesting that increased levels of translocated YopJ/P are directly responsible for increased death of DCs. Interestingly, although YopJn-GSK was also translocated at higher levels than YopJ-GSK, this was not enough to markedly enhance the level of cell death in DCs infected with YopJn-expressing bacteria (Figure 3D). This suggests that a fairly high threshold of YopJ activity is necessary to induce death of DCs, and that this threshold is significantly higher than for macrophages. Together, these results indicate that *Y. enterocolitica* has much greater levels of cytotoxicty toward DCs than *Y. pseudotuberculosis*, that this cytotoxicity is dependent upon the degree of YopP secretion and translocation, and that the cytotoxicity of *Y. pseudotuberculosis* toward DCs can be significantly increased by inducing high levels of YopJ translocation.

### Evolutionary selection for differential secretion of YopP and YopJ

**Figure 4:**
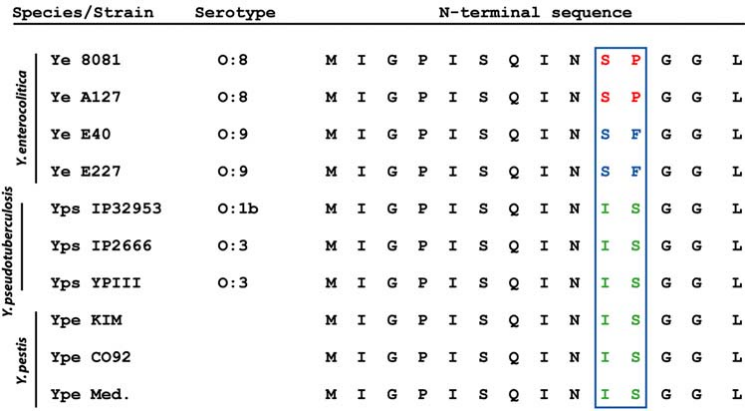
N-terminal sequence polymorphisms determine differential secretion of YopP/YopJ proteins. N-terminal 14 amino acids of YopP and YopJ from various *Yersinia* strains and serotypes. O∶8 serotypes of *Y. enterocolitica* possess an SP sequence at positions 10 and 11, whereas *Y. pseudotuberculosis* and *Y. pestis* possess the IS sequence. O∶9 serotypes of *Y. enterocolitica* also possess a polymorphism at amino acid 11, which likely accounts for the reduced secretion of YopP by O∶9 serotypes. Ye – *Y. enterocolitica*, Ype – *Y. pestis*, Yps – *Y. pseudotuberculosis*. Ype Med. – *Y. pestis*, biovar Medievalis.

*Y. enterocolitica* comprises a heterogeneous group of strains that differ with respect to their degree of virulence in animal infections. The primary basis for this difference is the presence or absence of a ‘High pathogenicity island’ (HPI) encoding an iron transport system [25]. Interestingly, the presence of the HPI shows strong correlation with the presence of arginine at amino acid 143 of YopP [27]. These two traits also correlate with apparently increased secretion of YopP, as the *Y. enterocolitica* serotypes which lack the HPI also appear to secrete less YopP [28]. Based on our analysis of the N-terminal polymorphisms between YopP from *Y. enterocolitica* 8081 and *Y. pseudotuberculosis* YopJ, we examined YopP sequences from strains of *Y. enterocolitica* O∶8 (high virulence) and O∶9 (low virulence) serotypes, as well as YopJ sequences from *Y. pestis*. We found that all sequenced strains of *Y. pestis* and *Y. pseudotuberculosis* possess the same isoleucine-serine sequence at positions 10 and 11, which correlates with reduced secretion (Figure 4). In contrast, O∶8 serotype, high virulence, strains of *Y. enterocolitica* possess the serine-proline sequence which confers high secretion. Interestingly, the O∶9 serotype low virulence *Y. enterocolitica* strains also possessed a mutation at position 11, encoding a phenylalanine rather than serine found in YopP from high virulence strains (Figure 4). YopP from the low virulence O∶9 strains has been suggested to be secreted at lower levels than YopP expressed by O∶8 serotype strains [28]. This raised the possibility that secretion of YopJ/YopP itself is a selectable trait that may play a differential role in virulence depending on the particular *Yersinia* species and serotype in which it is expressed. In order to examine this possibility in greater detail, we investigated the consequences of animal infection caused by *Y. pseudotuberculosis* expressing YopP or YopJ.

### YopP mediates greater cell death than YopJ in tissues during in vivo infection

**Figure 5:**
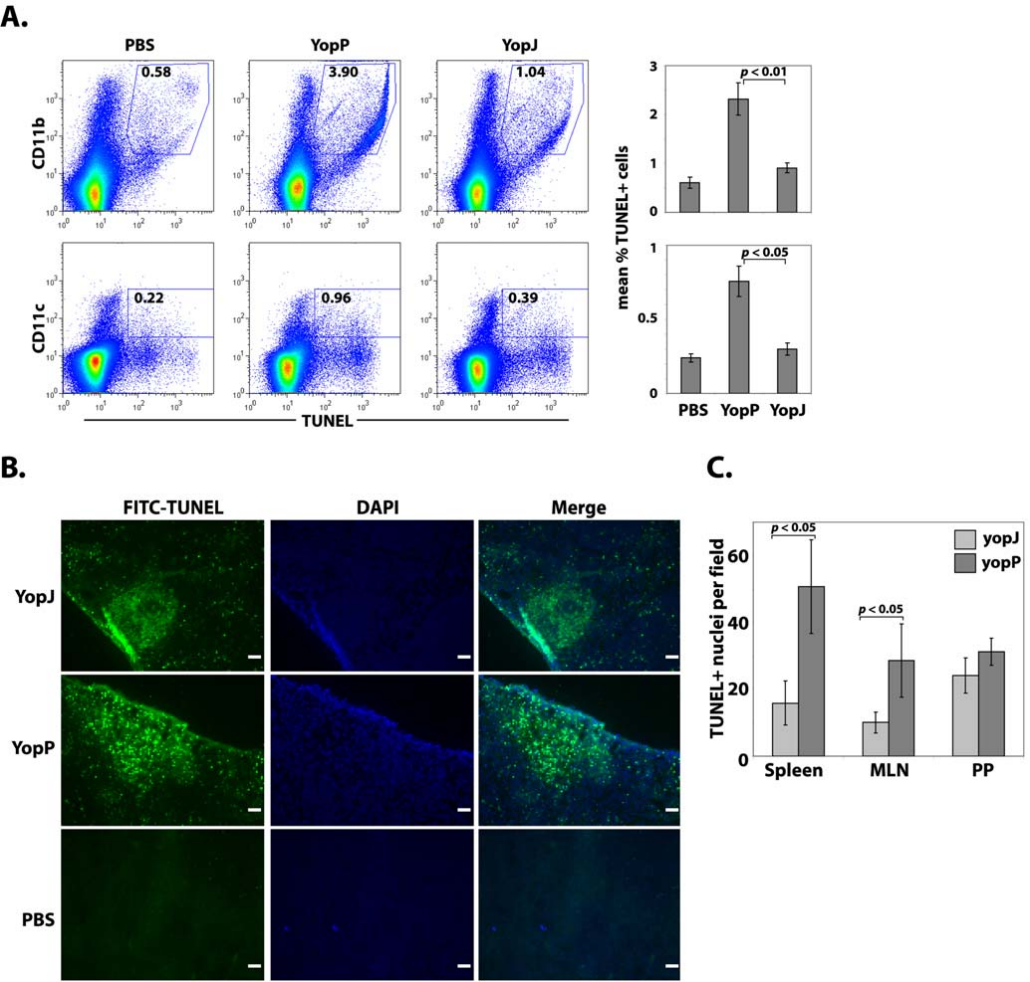
YopP-expressing *Y. pseudotuberculosis* causes enhanced killing of cells within lymphoid tissue during mouse infection. (A) TUNEL staining analyzed by flow cytometry of mesenteric lymph node cells isolated day 4 post-infection from mice given PBS as controls or infected with *yopJ* mutant *Y. pseudotuberculosis* expressing YopJ or YopP. CD11b and CD11c populations represent primarily macrophage and dendritic cell populations, respectively. Data are representative of six mice per group for infected mice and three mice for PBS controls. Percentage of double positive cells is indicated in each dot plot. Bar graphs represent mean percentage of double positive cells for six infected mice for each bacterial strain or three PBS control treated mice. (B) Spleen sections from mice infected with YopJ- or YopP- expressing bacteria as in (A), isolated on Day 4 post-infection and stained with TUNEL reagent and DAPI. Scale bar = 40 µM. (C) Enumeration of TUNEL^+^ nuclei from spleen, mesenteric lymph node, and Peyer's patch sections isolated four days post-infection with YopJ- (light grey bars) or YopP- (dark grey bars) expressing *yopJ* mutant *Y. pseudotuberculosis*. Data are averages of at least 10 random fields per mouse per tissue averaged from three mice per group. Similar results were obtained for two independent infections. *p* values were calculated using the unpaired Student's t-test.

Both *Y. enterocolitica* and *Y. pseudotuberculosis* cause cell death within infected tissues in vivo. Yet, a direct side-by-side comparison between the two species during in vivo infection is difficult due to unique gene products present or absent in one or the other species. We therefore directly examined the in vivo consequences of modulating *Y. pseudotuberculosis* cytotoxicity: we infected mice orally with either YopJ- or YopP-expressing *Y. pseudotuberculosis* and assayed the extent of cell death in infected tissues by terminal dUTP nick-end labeling (TUNEL) staining four days post-infection. Consistent with our in vitro observations, mice infected with the YopP-expressing strain of *Y. pseudotuberculosis* showed markedly increased levels of TUNEL-positive cells in mesenteric lymph nodes among both CD11b^+^ (primarily macrophage) and CD11c^+^ (primarily dendritic cell) populations (Figure 5A). B220^+^ (primarily B cell) populations also showed increased levels of TUNEL staining in mesenteric lymph nodes of mice infected with YopP-expressing bacteria relative to mice infected with YopJ-expressing bacteria (data not shown). This suggests that in vivo, multiple cell subsets are susceptible to *Yersinia*-induced cell death. Further microscopic examination also suggested than in mice infected with YopP-expressing bacteria, cell death was more widely distributed within the infected tissues, whereas in mice infected with YopJ-expressing bacteria, dead or dying cells were more tightly clustered within a smaller area (Figure 5B). In order to further corroborate and quantify these findings, we enumerated TUNEL^+^ cells in infected tissue sections. Significantly more TUNEL^+^ cells were present in the spleen and lymph node sections of mice infected with YopP-expressing bacteria than in the mice infected with YopJ-expressing bacteria, although no significant differences were observed in the Peyer's patches (Figure 5B). In vivo, higher numbers of CD11b^+^ cells were TUNEL^+^ in YopP-infected mice than YopJ-infected mice despite the observation that bone marrow derived macrophages were equally susceptible to death following infection with YopP- or YopJ-expressing bacteria (Figure 4B). However, we have observed that at low multiplicities of infection, YopP-expressing bacteria are more cytotoxic than YopJ-expressing bacteria to bone marrow derived macrophages as well (Figure S2). This may suggest that the actual multiplicity of infection within infected tissues is likely to be fairly low, even at later timepoints post-infection. An alternative possibility is that macrophages in vivo may behave slightly differently with respect to *Yersinia*-induced cell death than bone marrow derived macrophages in culture.

### Increased cytotoxicity attenuates in vivo virulence of *Y. pseudotuberculosis*

**Figure 6:**
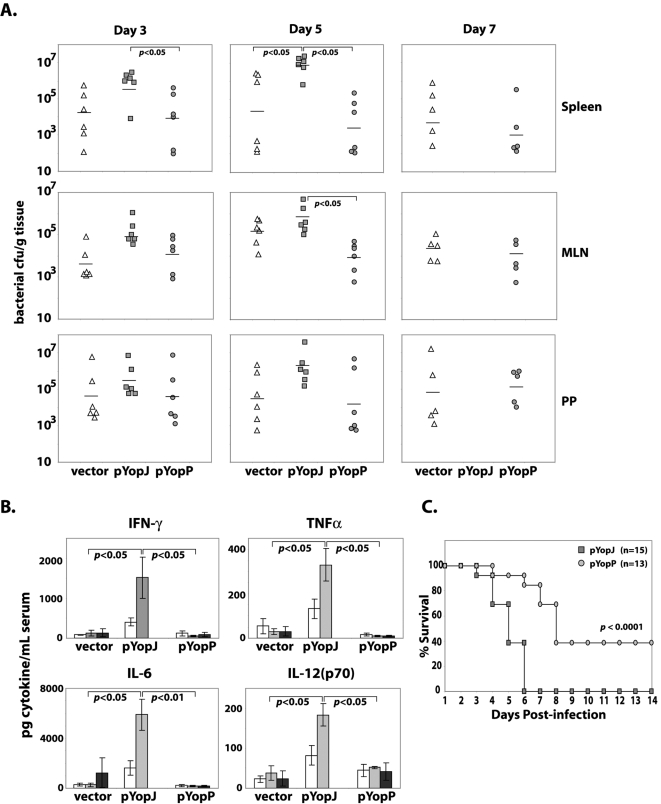
YopP-expressing *Y. pseudotuberculosis* is attenuated and induces lower levels of serum cytokine production during mouse infection. (A) Bacterial CFUs present in tissues of mice infected with Yp Δ*yopJ* containing either empty vector (white triangles) or expressing YopJ (grey squares) or YopP (grey circles) at days 3, 5, and 7 after oral infection. Mice infected with YopJ-expressing Yp Δ*yopJ* were moribund or dead from overwhelming infection at the end of day 6. (B) Serum cytokine levels of infected mice from (A) collected on days 3 (white bars), 5 (light grey bars), and 7 (dark grey bars). (C) Survival curve of mice infected with YopJ- (grey squares) or YopP- (grey circles) expressing Yp Δ*yopJ*. *p* values for bacterial cfu and serum cytokine levels were calculated using unpaired 2-tailed Student's t-Test. *p* value for mouse survival curves was calculated using log-rank test.

We investigated the impact of this increased cytotoxicity on the virulence of *Y. pseudotuberculosis* by examining bacterial growth in tissues of mice infected with the *yopJ* mutant strain reconstituted with either YopJ or YopP. Surprisingly, we found that the more cytotoxic (YopP-expressing) *Y. pseudotuberculosis* strain was attenuated following oral infection: mice infected with bacteria expressing YopP had significantly lower colony forming units (cfu) in the spleen at days 3 and 5 post-infection, and in mesenteric lymph nodes on day 5 post-infection (Figure 6A). Due to the acute nature of the infection, not enough mice infected with YopJ-expressing bacteria survived to be able to isolate tissues at day 7. The *Y. pseudotuberculosis* strain containing only the vector, and therefore completely lacking YopJ, was as deficient as the YopP-expressing strain for replicating in the spleen, consistent with the requirement of YopJ for this aspect of *Yersinia* virulence [23] (Figure 6A). Importantly, 97–100% of bacteria recovered from mouse tissues maintained the pYopJ or pYopP plasmids for at least seven days following oral infection, and the YopP-expressing bacteria maintained their increased in vitro cytotoxicity upon reisolation from infected organs (data not shown).

Levels of pro-inflammatory cytokines were markedly elevated in sera obtained from mice infected with YopJ-expressing bacteria compared with those infected either with YopP-expressing bacteria or the isogenic *yopJ*-deficient bacteria containing only vector (Figure 6B). Elevated levels of TNF-α and IL-6 were observed on day 3 post-infection, and rose dramatically along with IFN-γ and IL-12 on day 5, coincident with the increase in bacterial load in the spleen. Surprisingly, lower levels of cytokines were observed in mice infected with either YopP-expressing bacteria or bacteria lacking *yopJ* (vector), despite the ability of the mice to limit the systemic replication of these strains. Furthermore, survival of mice infected with YopP-expressing bacteria was markedly greater than that of mice infected with bacteria expressing YopJ (Figure 6C). This indicated that excessive cytotoxicity was detrimental to *Y. pseudotuberculosis* replication and virulence in vivo. We obtained similar results in infection of mice with bacteria containing the pYopPJn plasmid (Figure S3). Although this strain hyper-secretes YopJ and is more cytotoxic in vitro than wild-type *Y. pseudotuberculosis* (Figure 3), it too is attenuated in vivo as measured by survival of infected mice (Figure S3). These data indicate that limiting cytototoxicity during infection through control of YopJ secretion is critical for optimal virulence of *Y. pseudotuberculosis*.

## Discussion

Successful infection of mammalian organisms by microbial pathogens is often associated with disruption of cell death homeostasis within infected tissues. Triggering of host-cell death is thought to be an important virulence characteristic of many facultative pathogenic bacteria, since deletion of individual bacterial genes or secretion systems associated with causing cell death leads, in many cases, to attenuation of virulence in experimental infection models [1]. However, it is likely that evolution of any given microbial pathogen has led to an optimized set of virulence traits in which any particular characteristic, such as the ability to cause cell death, is tightly regulated in relation to the other traits. We examined this hypothesis by using a system in which the degree of cytotoxicity could be modulated. We chose *Yersinia* as a model system, because, despite the close evolutionary relatedness of the pathogenic *Yersiniae*, they are heterogeneous in the extent of cell death and pathology that they cause during infection [6],[27]. This provides optimal conditions for testing whether the degree of cell death caused by a particular member of a related group of organisms might be an evolutionarily selected trait.

Recent studies have shown that two of the pathogenic *Yersinia* species, *Y. enterocolitica* and *Y. pestis*, possess varying degrees of cytotoxicity, and that *Y. enterocolitica* YopP is translocated more efficiently into cells than the homologous protein YopJ of *Y. pestis* [29]. These studies also demonstrated that expressing YopP from a strong promoter on a high copy plasmid in *Y. pestis* enhanced the ability of *Y. pestis* to cause macrophage cell death in vitro. Variation also exists among different serotypes of *Y. enterocolitica* with respect to both YopP activity [27] and degree of secretion [28]. However, nothing is currently known about the contribution of differential YopJ/YopP secretion to cytotoxicity or virulence of *Yersinia* during in vivo infection. Furthermore, the underlying basis for the differential secretion of YopP and YopJ, as well as differential secretion of YopP among individual *Y. enterocolitica* isolates, remains unclear.

The work presented here demonstrates that YopP and YopJ are differentially secreted and translocated due to a polymorphism in amino acids 10 and 11 between *Y. pseudotuberculosis* YopJ and *Y. enterocolitica* YopP. Polymorphisms at amino acid 11 are also likely to account for differences in secretion of YopP among different *Y. enterocolitica* serotypes. However, these amino acids are identical among YopJ proteins from sequenced strains of *Y. pseudotuberculosis* and *Y. pestis*. This suggests that selection pressures driving this difference in secretion between the *Y. enterocolitica* and *Y. pseudotuberculosis* proteins were maintained in the recent evolution of *Y. pestis* from *Y. pseudotuberculosis*. It is interesting to note that while *Y. pestis* YopJ contributes to host cell death and blocks cytokine secretion in vitro, as it does for enteric *Yersiniae*, it appears to be nonessential for virulence in a rat model of bubonic plague [45]. Our study suggests that expression of YopP or a hypersecreted form of YopJ in *Y. pestis* may also attenuate virulence during in vivo infection. Our data also indicate that despite inducing higher levels of cell death in infected tissues (Figure 5), *Y. pseudotuberculosis* expressing YopP were attenuated for colonizing and possibly replicating within the spleen and mesenteric lymph nodes (Figure 6). An intermediate level of YopJ protein delivery therefore appears necessary for maximal virulence, as bacteria completely lacking YopJ are also deficient in colonization of the spleen following oral infection, consistent with earlier studies [23]. Recent work indicates that YopJ is not required for replication of *Y. pseudotuberculosis* in the spleen if the bacteria are delivered intraperitoneally, bypassing the intestinal route [46]. Our results therefore indicate a requirement for intermediate levels of YopJ delivery in mediating spread of bacteria to the spleen from initial sites of colonization within the intestine.

The underlying basis for why increasing cytotoxicity of *Y. pseudotuberculosis* infection leads to attenuation are not clear at this time, but could involve an intracellular stage during in vivo infection [47],[48]. Excessive cytotoxicity may lead to death of cells that provide an important intracellular niche for *Y. pseudotuberculosis*, potentially for purposes of spreading from the initial site of colonization to other tissues. Recent evidence indicates that spread of *Y. pseudotuberculosis* to the spleen following oral infection occurs directly from a replicating pool within the intestinal lumen, through a pathway that bypasses the mesenteric lymph nodes [49]. Interestingly, despite the differential susceptibility of bone-marrow derived macrophages and dendritic cells to YopJ-mediated killing (Figures 1 and 3), it appears that at least within mesenteric lymph nodes, both CD11b^+^ (primarily macrophage) and CD11c^+^ (primarily dendritic) cell populations showed either high or intermediate levels of cell death when infected by YopP- or YopJ- expressing *Y. pseudotuberculosis* (Figure 5). A particular phagocytic cell subset within the Peyer's patches or intestinal lamina propria may be responsible for transporting *Yersinia* from the intestine to systemic sites, and these cells may be differentially susceptible to YopJ-mediated cell death. Recent work has highlighted the role of a particular subset of dendritic cells within the lamina propria, designated CX_3_CR1^+^ cells, in uptake of *Salmonella typhimurium* [50],[51]. Investigation of the particular cell types that interact with *Yersinia* during intestinal colonization and the utilization of mouse models enabling dendritic cell depletion are likely to provide further insight into the mechanism of *Yersinia* spread to internal tissues.

That higher in vitro cytotoxicity of *Y. pseudotuberculosis* can lead to markedly reduced virulence is somewhat surprising, particularly since we observed reduced cytokine production as well as increased cell death in mice infected with the more cytotoxic, YopP-expressing bacteria. An alternative hypothesis for the requirement for intermediate levels of cytotoxicity during *Y. pseudotuberculosis* infection is that proinflammatory cytokines produced during wild-type *Y. pseudotuberculosis* infection could contribute to bacterial spread by promoting tissue destruction. This could be blocked during infection with YopP-expressing *Y. pseudotuberculosis* due to death of cytokine-producing cells. In support of this model, recent work has shown that mice infected with the attenuated Δ*pla* strain of *Y. pestis* control the infection without producing high levels of cytokines in contrast to wild-type infected mice, which fail to control the infection despite high levels of cytokine production [52]. These observations may reflect the possibility that appropriate control of bacterial replication must occur very early during the infection process. The outcome of a bacterial infection depends on the interplay between host clearance mechanisms and bacterial virulence factors; misregulation of bacterial virulence factors as we observed with expression of YopP in place of YopJ therefore appears to shift the balance strongly in favor of the host.

In addition to implications for the evolution of bacterial virulence, this work sheds further light on mechanisms of Type III secretion in *Yersinia*. It has been suggested that mRNA sequence determines the secretion of TTSS effectors [40]. An alternative proposal is that the amphipathic character of the N-terminal amino acids controls the extent of secretion of TTSS effectors [42]; this latter study further demonstrated that seemingly minor changes in the amino acid sequence of a synthetic secretion signal could result in dramatically different levels of secretion of a reporter fusion construct. In agreement with this study, we demonstrate that in the case of YopJ/P, the amino acid sequence plays a key role in controlling secretion. It may be that alteration of IS to SP at positions 10 and 11 generates a more optimal amphipathic sequence leading to enhanced secretion and translocation of YopP relative to YopJ. There may nonetheless be a contribution from the mRNA sequence that is not revealed directly by these studies. Indeed, the replacement of *yopJ* noncoding sequences with those of *yopP* to generate YopPJn from YopJn led to markedly higher translocation of the corresponding GSK fusion protein and secretion of the full length protein (Figure 3B and Figure S1). We further observed that YopE was also secreted at higher levels by *Y. enterocolitica* than *Y. pseudotuberculosis* (Figure 4A). However, the *yopE* coding sequences are identical between the two bacterial species. This suggests that the rules governing the secretion of proteins that possess dedicated chaperones (such as YopE) may differ from those that do not (such as YopJ/P). Individual type III effector proteins may therefore differ in the degree to which their secretion is governed by mRNA or primary amino acid sequences.

It is notable that while bacteria secreting either YopJ or YopP show cytotoxicity toward macrophages, as previously described [16],[17], only bacteria secreting YopP or hypersecreted mutants of YopJ were cytotoxic toward DCs (Figures 1 and 3). This potent cytotoxicity exhibited by *Y. enterocolitica* toward DCs may be a confounding factor in the interpretation of some of the data describing the inhibition of particular aspects of DC biology such as maturation and phagocytosis [30],[32]. We did not observe reduced maturation of, or inhibition of cytokine secretion by DCs infected by *Y. pseudotuberculosis*, in contrast to *Y. enterocolitica* infected DCs (Figure S4). A recent study indicates, however, that YopJ of *Y. pestis* can inhibit differentiation of monocytes into DCs and also interferes with the ability of DCs to stimulate T cell proliferation [53]. Many of these studies were performed with a monocytic cell line which can be differentiated in vitro into DC-like cells or with human monocytes isolated from peripheral blood samples. Dendritic cells *in vivo* are not a uniform cell population, but rather comprise a diverse array of closely related cells that differ in their activation and antigen-presentation properties [54],[55]. Furthermore, murine and human DC populations also differ with respect to their activation properties and expression of TLR molecules [56]. Experimental differences observed with different systems may reflect potential differences between DC subsets in vivo. Different subsets of DCs may therefore exhibit different maturation or survival properties when targeted by *Yersinia* during in vivo infection.

Macrophage resistance to cell death in response to inflammatory stimuli such as LPS or bacterial infection is mediated by both NF-κB and p38 MAPK-dependent transcription of a variety of anti-apoptotic proteins [57],[58]. Inhibition of both NF-κB and MAPK signaling in the context of *Yersinia* infection is required for the full extent of *Yersinia*-induced macrophage cell death [36]. Interestingly, pretreatment of DCs with the p38 MAPK inhibitor SB202190 was sufficient to raise the level of DC death following *Y. pseudotuberculosis* infection to that of *Y. enterocolitica* infected cells (Figure 2D). We did not observe differences in the ability of *Y. pseudotuberculosis* and *Y. enterocolitica* to inhibit IκB-α degradation in either macrophages or DCs (data not shown). These findings indicate that DCs and macrophages are differentially susceptible to host cell death induced by *Yersinia* infection, and this may depend on differential inhibition of MAPK pathways or differential regulation of MAPK-dependent genes in macrophages and dendritic cells during *Yersinia* infection. Data corroborating these observations have also recently been reported by Adkins et al. [59].

### Conclusion

Our work uncovers a previously unappreciated feature of YopJ-dependent cell death in the context of *Yersinia* infection. Specifically, we have shown that this virulence property must be carefully balanced during infection by *Y. pseudotuberculosis*, and that shifting this balance in a way that leads to increased cell death attenuates bacterial virulence. Similar findings have recently been described in the organism *Francisella novicida*: bacterial genes were identified that are necessary to tightly regulate the induction of macrophage death in vitro, and mutants lacking these genes were dramatically attenuated following mouse infection [60]. While this is reasonable in light of the *Francisella* intracellular lifestyle, it is surprising given the traditional viewpoint that the primary site of *Yersinia* replication is extracellular. Our work therefore highlights the importance of regulating host cell death during *Yersinia* infection, and indicates that the appropriate regulation of bacterially-induced host cell death may be a universal theme in the pathogenesis of bacterial infection.

## Materials and Methods

### Cell culture

Bone marrow was isolated from 6–8 week old C57Bl/6 mice and cultured in 12-well plates in DMEM containing 5% FCS in the presence of GM-CSF for dendritic cells (DCs) [61] or 10% FCS and 30% L929 cell supernatant for bone marrow macrophages (BMMs) [62]. Cells were maintained at 37°C in a 5% CO_2_ humidified incubater. DCs were fed on days 2 and 4 post-isolation and infected on day 5 post-isolation. BMMs were harvested and replated into 12 or 24-well plates on days 6–7 post-isolation and infected the next day.

### Bacterial strains and infection conditions

Strains used in this study are indicated in Table S1. *Y. pseudotuberculosis* strain IP2666 and isogenic mutants were a kind gift of Dr. James Bliska, SUNY Stonybrook. *Y. enterocolitica* strain 8081 was the gift of Dr. Denise Monack, Stanford University. Bacteria were routinely grown at 26°C. For infection of cultured cells, bacteria were grown shaking overnight at 26°C in 2×YT medium. Bacteria were diluted in 2×YT containing 20 mM MgCl_2_ and 20 mM sodium oxalate. Bacteria were grown shaking for 1 hour at 26°C followed by 37°C for 2 hours to induce Yop secretion [13]. Bacteria were harvested, washed three times with DMEM and resuspended at the appropriate density in DMEM before being added to cells. Bacteria were spun onto the cells at 1000 RPM for 5 minutes, and the infected cells placed in a humidified tissue culture incubator at 37°C for 1 hour. Gentamicin was added to the cells 1 hour post-infection to a final concentration of 100 µg/mL, and the cells placed in the incubator until harvesting. LPS used in indicated experiments was O55∶B5 obtained from Sigma (L2880).

### Plasmid constructions

Primers used to amplify YopJ were identical to those previously described [16]. A similar sized fragment containing both the ORF and flanking regions of YopP was amplified from *Y. enterocolitica* 8081 DNA using the following primers: Forward 5′-GAGAGAAAAGTTGCGAGAGCTG-3′. Reverse 5′-ACGTCGATATGTCATGTATAT-3′. Both YopJ and YopP amplified fragments were cloned into the SphI/EagI sites of pACYC184. pYopJP and pYopPJ were constructed by exchanging EcoNI/BstEII fragments that contained the YopJ and YopP ORFs with minimal flanking sequence from each of the parent plasmids. Site directed mutant constructs were generated by PCR-based mutagenesis using standard PCR conditions and primers containing altered base mutations. Multiple independently cloned constructs were sequenced prior to further use. GSK-tag fusion constructs were generated as follows: complimentary oligos (Invitrogen) containing the first 39 nucleotides of the GSK-3β coding sequence [44] flanked by XcmI and BstEII restriction sites were annealed and ligated into the XcmI/BstEII sites of pYopJ, pYopP, pYopJn, and pYopPJn plasmids.

### Cell Death Assay

Cells were harvested 6 hours post-infection or 18–20 hours post-infection as indicated, and washed twice with Annexin V staining buffer (10 mM HEPES, 140 mM NaCl, 2.5 mM CaCl_2_) before being stained with Annexin V (Molecular Probes) and propidium iodide (Sigma) (1 µg/mL final concentration) as described in the Vybrant apoptosis assay kit (Molecular Probes). Detection of caspase-3 activation was done with the NucView™ 488 Caspase-3 fluorescent substrate (Biotium, Inc.). Flow cytometry was performed on a FACSCalibur (Becton Dickinson) followed by analysis with FlowJo software (Treestar, Inc.).

### Western Blotting and Antibodies

Antibodies used were against cleaved PARP (R&D Systems) and phospho- and total-MAPKs (Cell Signaling Technologies). Antibodies against phospho-GSK-3β (Ser9) (#9336) and against GSK-3β tag (#9325) were from Cell Signaling Technologies. Secondary antibodies were horseradish peroxidase conjugated anti-rabbit or anti-mouse (Jackson Immunoresearch). BMMs and DCs were harvested 2 hrs post-infection for PARP cleavage and at indicated times for phospho-MAPK detection. Cells were lysed in buffer containing 50 mM Tris-HCl pH 8.0, 5 mM EDTA, 2% Triton X-100, 0.02% sodium azide with protease inhibitors. 1/8 of each cell lysate was run on 12.5% SDS-PAGE and transferred to PVDF membrane (Millipore Corporation) prior to Western blotting. Detection was with ECL reagent (Amersham/GE Healthcare).

### Secreted Yop Analysis

Bacteria were grown overnight and diluted into 2xYT containing 20 mM MgCl_2_ and 20 mM sodium oxalate as described above. Bacteria were grown for 1 hr. at 25°C and 6 hr. at 37°C. The OD_600_ of the cultures were assayed and bacteria were spun at 4000 RPM in a clinical centrifuge. Supernatants were collected and precipitated with 10% TCA, washed with acetone, air-dried and resuspended in SDS-PAGE loading buffer. The protein samples were normalized for the OD_600_, run on 10% SDS-PAGE gel and analyzed by staining with Coomassie blue.

### Analysis of YopJ/P translocation

The detection of translocated proteins was performed essentially as described [44]. Briefly, HeLa cells were seeded into 24 well dishes at a density of 3×10^5^ cells per well. Cells were infected the following day with a *yopJ* mutant of *Y. pseudotuberculosis* expressing YopJ_GSK_, YopP_GSK_, YopJn_GSK_, or YopPJn_GSK_ after first growing the bacteria in inducing conditions described above. 2 hours prior to infection, cells were washed once with DMEM without serum, and incubated in the same medium for the rest of the experiment. Bacteria were harvested, washed 3 times in DMEM without serum, and added to the cells at an MOI of 25. Bacteria were allowed to infect cells for three hours, after which the cells were washed once with PBS, and lysed in cell lysis buffer as described above. Cell lysates were run on 10% SDS-PAGE gels, transferred to PVDF membrane, and probed with polyclonal antibodies to phospho-GSK (Ser9). The blots were then stripped and reprobed with polyclonal antibodies to GSK tag (amino acids 1–13).

### Mouse Infections

8–10 week old female C57Bl/6J mice were infected with 5×10^8^ to 1×10^9^ bacteria in 0.1 mL PBS by intragastric inoculation using a feeding needle in accordance with Yale University approved animal protocols. Mice were fasted for 14–16 hours prior to infection. At indicated times post-infection, mice were sacrificed by CO_2_ asphyxiation and the indicated organs harvested, homogenized in PBS, and dilutions plated onto LB containing 2 µg/mL irgasan. Blood was collected from infected mice just prior to sacrificing by retro-orbital bleeding and centrifuged to collect serum. Serum cytokines were assayed using the Luminex 200 instrument (Luminex Corp.) in combination with the Beadlyte® Multicytokine Detection System 2 (Millipore).

### TUNEL staining

Tissues from infected mice were isolated and cryopreserved in OCT compound for sectioning or strained through nylon mesh to generate single-cell suspensions for staining and flow-cytometry analysis. For analysis of whole tissues, 10–12 µM thick sections were cut, air-dried, and fixed in 4% paraformaldehyde, followed by permeabilization in 0.1% citrate/0.1% Triton X-100. For FACS analysis, tissues were stained with appropriate antibodies (BD-Pharmingen) and fixed in 2% paraformaldehyde followed by permeabilization as above. Following fixation and permeabilization, tissues were stained with TUNEL reagent (Roche Applied Science) according to manufacturer's instructions and either visualized by microscopy using a AxioPhot 2 Micropscope with AxioVision software (Zeiss) or analyzed by flow cytometry as described above. Single-stained and unstained samples were used for compensation controls in accordance with standard FACS procedures. TUNEL^+^ populations were determined by gating on cells present in TUNEL-stained samples that were absent in non-TUNEL samples.

## Supporting Information

Figure S1 N-terminal amino acid polymorphisms modulate secretion levels of YopJ and YopP. (A) Schematic diagram of YopJ and YopP constructs described in this work. YopJ and YopP open reading frames were exchanged by digestion of pYopJ and pYopP with EcoNI and BstEII and replacing the coding sequences of YopJ/P from one vector with that of the other. (B) Alignment of N-terminal region of *yopJ* and *yopP* genes with encoded amino acid sequence. The non-coding sequences between the EcoNI site and the translation start site are indicated in lower case. The EcoNI site is underlined. (C) Sequence of YopJ_n_, YopJ_2nt_ and YopJ_2aa_ mutant constructs. (D) TCA precipitated supernatants from indicated bacterial cultures grown in low calcium medium and analyzed by SDS-PAGE.
(15.17 MB PNG)

Figure S2 Low MOI infection of bone marrow derived macrophages reveals difference in extent of cell death caused by *Y. enterocolitica* and *Y. pseudotuberculosis*. Bone marrow derived macrophages were infected with MOI of 5 with indicated bacterial strains and assayed for annexin V and propidium iodide staining 18–20 hours post-infection.
(1.60 MB TIF)

Figure S3 Hypersescretion of YopJ attenuates *Y. pseudotuberculosis* virulence. Mice were infected orally with 5×10^8^ cfu of indicated bacterial strains and percent survival over time post-infection was analyzed.
(1.32 MB TIF)

Figure S4 Maturation of dendritic cells infected by wild-type *Y. pseudotuberculosis* but not *Y. enterocolitica* or *Y. pseudotuberculosis* expressing YopP. (A) CD40 and CD86 surface staining on DCs 18–20 hours post-infection with indicated bacterial strains. CD40 and CD86 are upregulated on DCs treated with LPS or wild-type and plasmid-cured *Y. pseudotuberculosis* as well as plasmid-cured *Y. enterocolitica*. No upregulation is observed on DCs infected with wild-type *Y. enterocolitica* or *Y. pseudotuberculosis* expressing YopP. (B) Secretion of IL-6 and IL-12 into DC culture supernatant was assayed 18 hours post-infection. No cytokines are detectable in culture supernatants from cells infected with *Y. enterocolitica*. The presence of the *Yersinia* virulence plasmid does not appear to inhibit cytokine production in DCs infected with wild-type *Y. pseudotuberculosis*.
(1.87 MB TIF)

Table S1 Strains and plasmids used in this study.
(0.06 MB DOC)

Table S2 Percent of cells containing β-lactamase activity after infection with *Y. pseudotuberculosis* expressing YopE-BlaM fusion protein.
(0.03 MB DOC)
