# Supplementary figures and images for: Reduced Secretion of YopJ by Yersinia Limits In Vivo Cell Death but Enhances Bacterial Virulence
Source: PLoS Pathog. 2008 May 16;4(5):e1000067. doi: 10.1371/journal.ppat.1000067 (PMC2361194; doi:10.1371/journal.ppat.1000067)

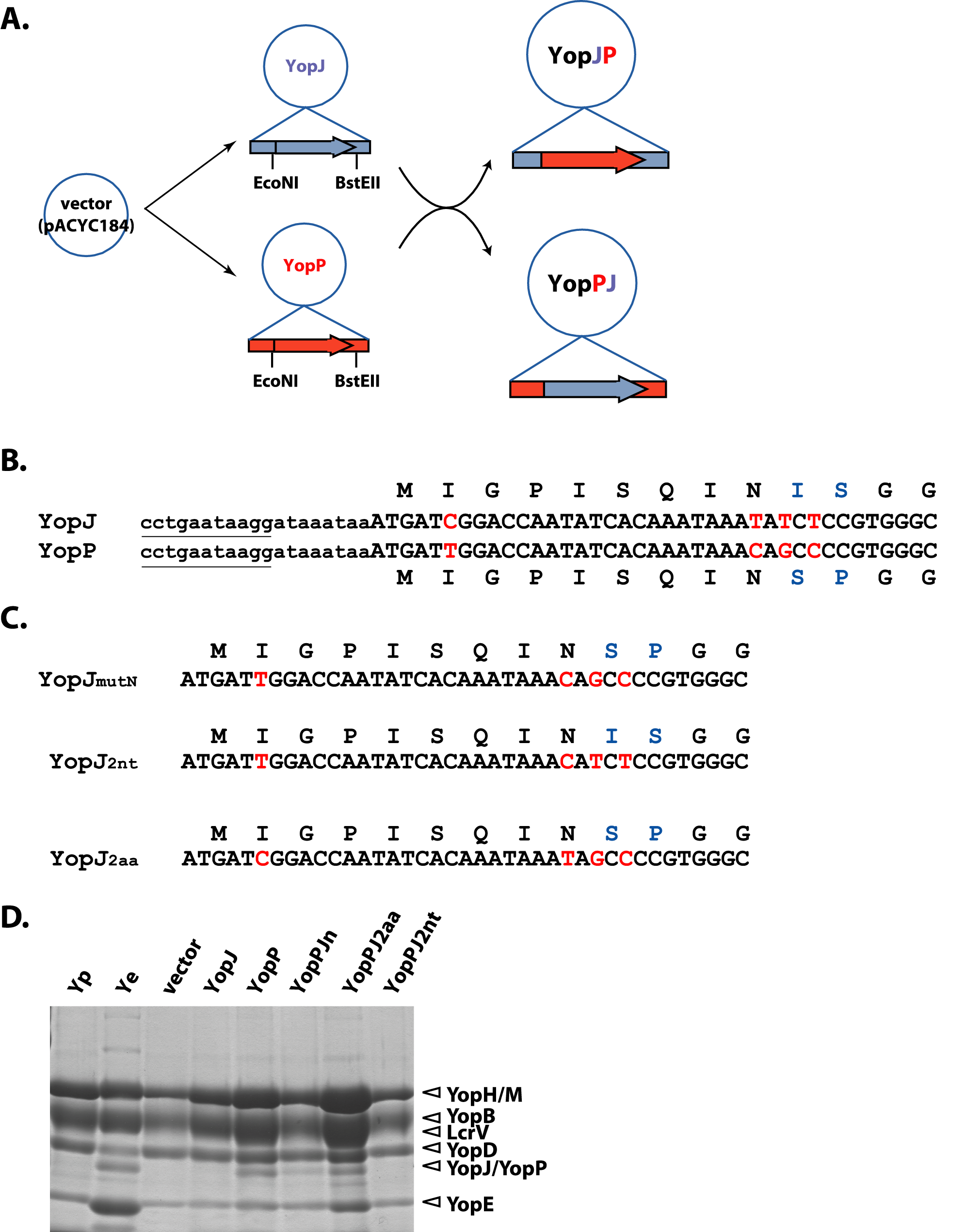

Supplement: Figure S1 — N-terminal amino acid polymorphisms modulate secretion levels of YopJ and YopP. (A) Schematic diagram of YopJ and YopP constructs described in this work. YopJ and YopP open reading frames were exchanged by digestion of pYopJ and pYopP with EcoNI and BstEII and replacing the coding sequences of YopJ/P from one vector with that of the other. (B) Alignment of N-terminal region of yopJ and yopP genes with encoded amino acid sequence. The non-coding sequences between the EcoNI site and the translation start site are indicated in lower case. The EcoNI site is underlined. (C) Sequence of YopJn, YopJ2nt and YopJ2aa mutant constructs. (D) TCA precipitated supernatants from indicated bacterial cultures grown in low calcium medium and analyzed by SDS-PAGE. (15.17 MB PNG) [file ppat.1000067.s001.png]

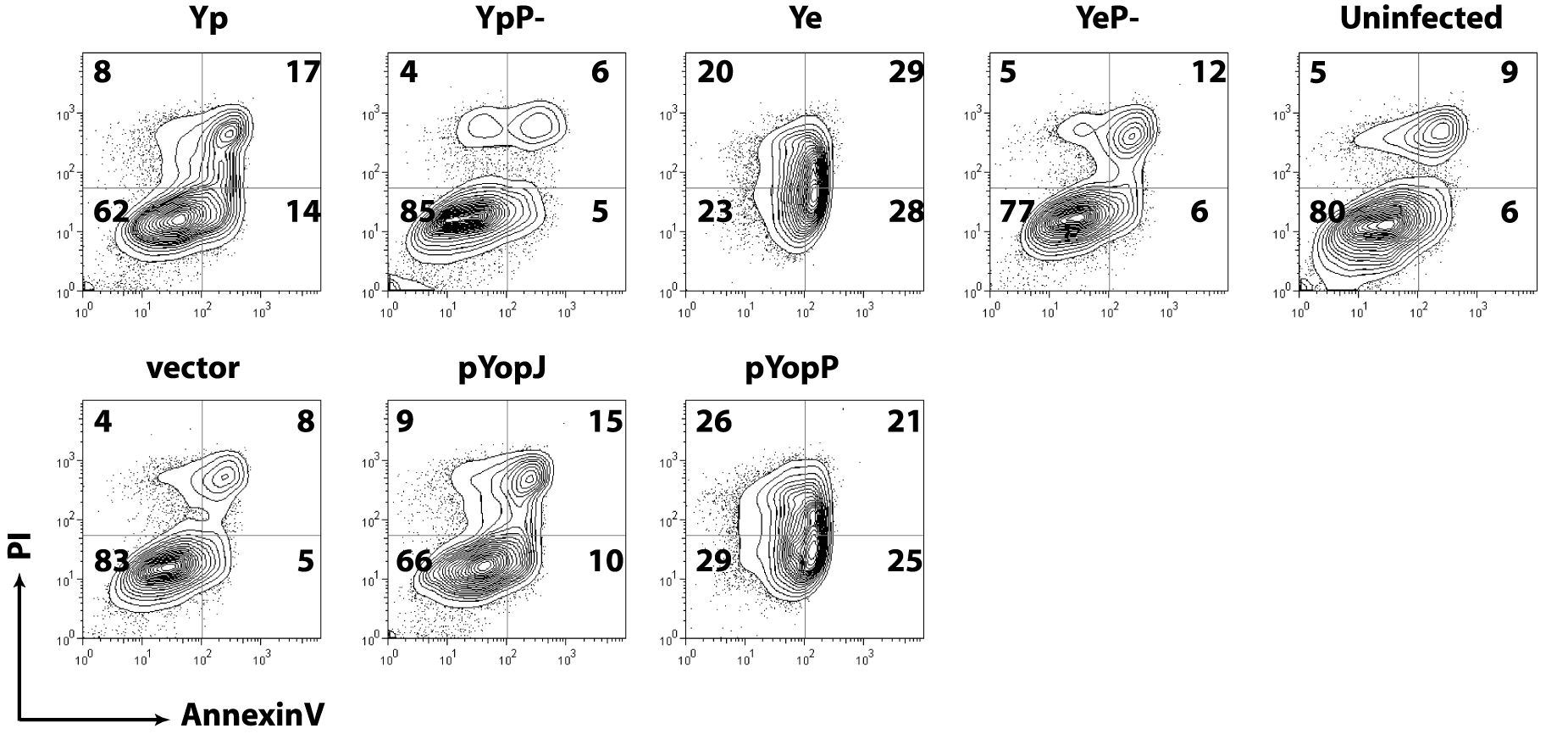

Supplement: Figure S2 — Low MOI infection of bone marrow derived macrophages reveals difference in extent of cell death caused by Y. enterocolitica and Y. pseudotuberculosis. Bone marrow derived macrophages were infected with MOI of 5 with indicated bacterial strains and assayed for annexin V and propidium iodide staining 18–20 hours post-infection. (1.60 MB TIF) [file ppat.1000067.s002.tif]

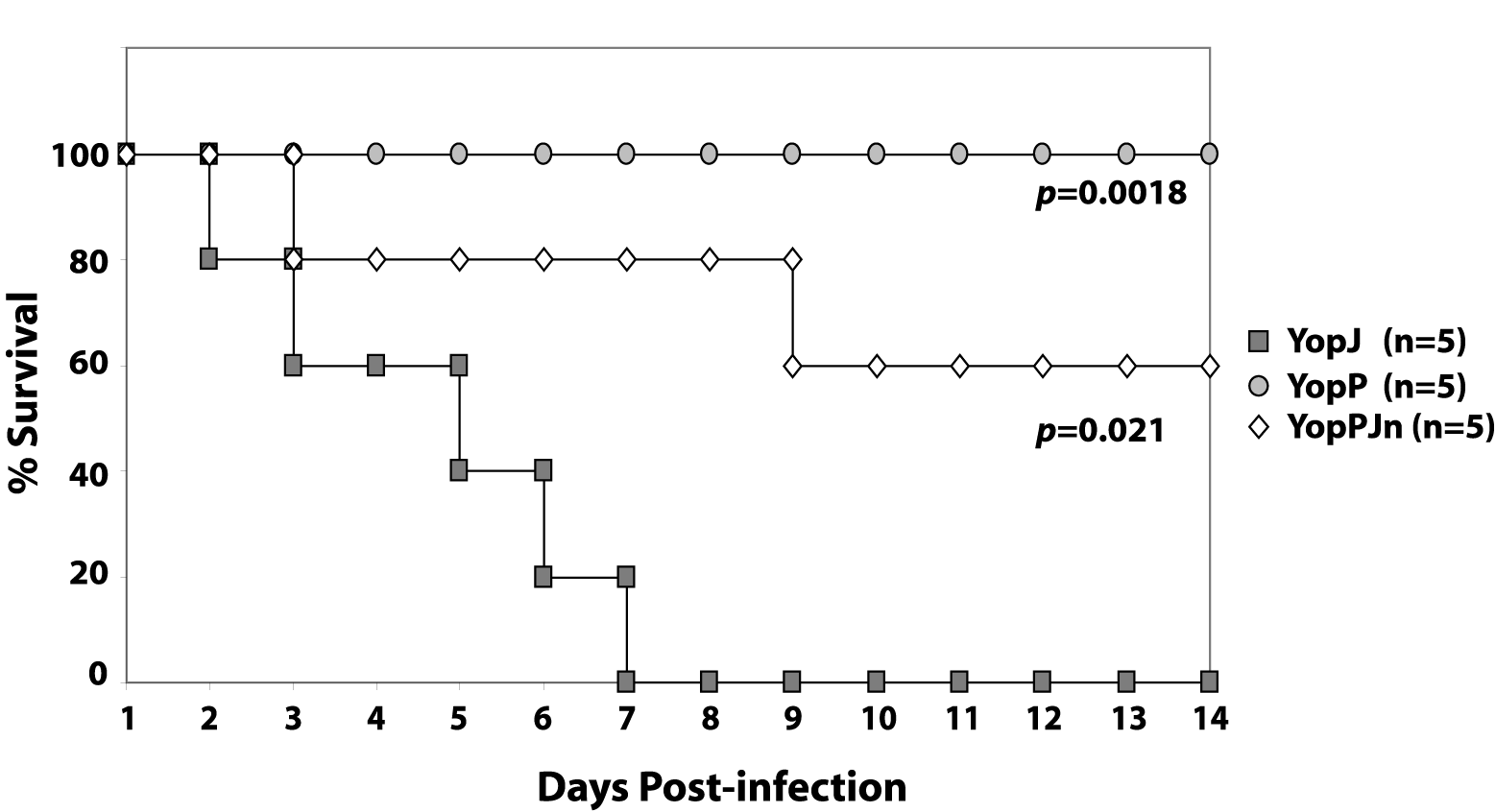

Supplement: Figure S3 — Hypersescretion of YopJ attenuates Y. pseudotuberculosis virulence. Mice were infected orally with 5×10^8^ cfu of indicated bacterial strains and percent survival over time post-infection was analyzed. (1.32 MB TIF) [file ppat.1000067.s003.tif]

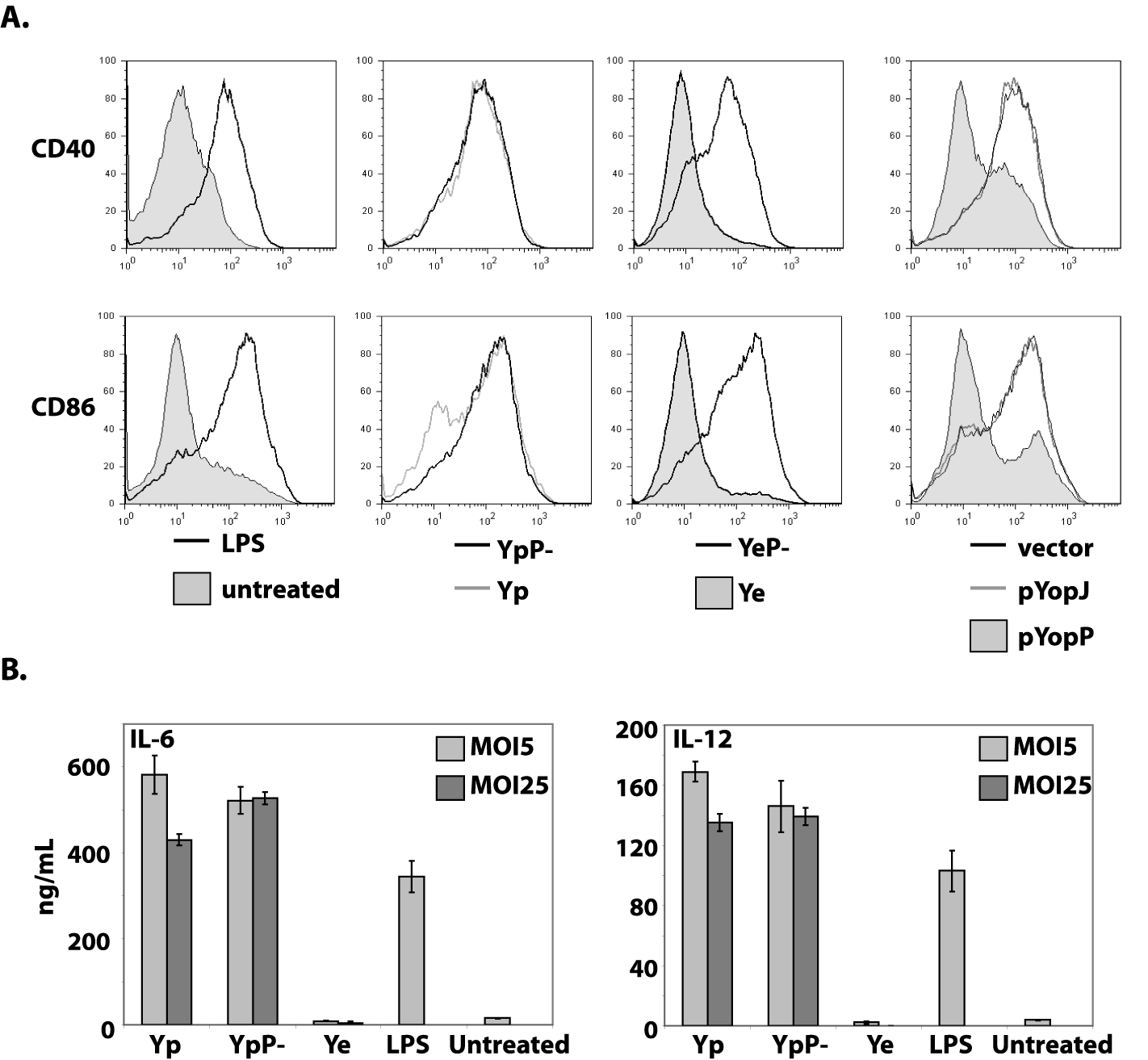

Supplement: Figure S4 — Maturation of dendritic cells infected by wild-type Y. pseudotuberculosis but not Y. enterocolitica or Y. pseudotuberculosis expressing YopP. (A) CD40 and CD86 surface staining on DCs 18–20 hours post-infection with indicated bacterial strains. CD40 and CD86 are upregulated on DCs treated with LPS or wild-type and plasmid-cured Y. pseudotuberculosis as well as plasmid-cured Y. enterocolitica. No upregulation is observed on DCs infected with wild-type Y. enterocolitica or Y. pseudotuberculosis expressing YopP. (B) Secretion of IL-6 and IL-12 into DC culture supernatant was assayed 18 hours post-infection. No cytokines are detectable in culture supernatants from cells infected with Y. enterocolitica. The presence of the Yersinia virulence plasmid does not appear to inhibit cytokine production in DCs infected with wild-type Y. pseudotuberculosis. (1.87 MB TIF) [file ppat.1000067.s004.tif]
