# Supplementary material for: Reduced Secretion of YopJ by Yersinia Limits In Vivo Cell Death but Enhances Bacterial Virulence
Source: PLoS Pathog. 2008 May 16;4(5):e1000067. doi: 10.1371/journal.ppat.1000067 (PMC2361194; doi:10.1371/journal.ppat.1000067)
Supplement: Table S1 — Strains and plasmids used in this study. (0.06 MB DOC) [file ppat.1000067.s005.doc]

**Table S1. Strains and Plasmids used in this study**.

**Strain/Plasmid Description Reference or Source**

| IP2666 (Yp) | Wild-type *Y. pseudotuberculosis*, serogroup O:3 | J. Bliska |
| --- | --- | --- |
| IP2666c (YpP-) | IP2666 virulence plasmid-cured | J. Bliska |
| 8081 (Ye) | Wild-type Y. enterocolitica, serotype O:8 | Portnoy et al. 1984 |
| 8081c (YeP-) | 8081 virulence plasmid-cured | Portnoy et al. 1984* |
| IP26 (YpΔ*yopJ*) | IP2666 *yopJ* mutant | J. Bliska |
| pACYC184 | p15A Ori, Cm^r^, Tc^r^ | Chang and Cohen 1978 |
| pYopJ | pACYC184-containing 2000bp *yopJ* fragment cloned into SphI/EagI sites | This work |
| pYopP | pACYC184-containing 1920bp *yopP* fragment cloned into SphI/EagI sites | This work |
| pYopJP | pYopJ containing EcoNI/BstEII fragment from pYopP | This work |
| pYopPJ | pYopP containing EcoNI/BstEII fragment from pYopJ | This work |
| pYopJn | pYopJ with 4 point mutations are 5’ end of *yopJ* coding sequence | This work |
| pYopPJn | pYopPJ with 4 point mutations at 5’ end of *yopJ* coding sequence | This work |
| pYopPJ2aa | pYopPJ with 2 non-synonmous point mutations at 5’ end of *yopJ*. | This work |
| pYopPJ2nt | pYopPJ with 2 synonomous point mutations at 5’ end of *yopJ* | This work |

* Independently derived during these studies from 8081 strain by growth at 37 ºC and plating on Congo red-containing agar plates.
