# Supplementary material for: Reduced Secretion of YopJ by Yersinia Limits In Vivo Cell Death but Enhances Bacterial Virulence
Source: PLoS Pathog. 2008 May 16;4(5):e1000067. doi: 10.1371/journal.ppat.1000067 (PMC2361194; doi:10.1371/journal.ppat.1000067)
Supplement: Table S2 — Percent of cells containing β-lactamase activity after infection with Y. pseudotuberculosis expressing YopE-BlaM fusion protein. (0.03 MB DOC) [file ppat.1000067.s006.doc]

**Table S2.** Percent of cells containing β-lactamase activity after infection with *Y. pseudotuberculosis* expressing YopE-BlaM fusion protein

% cells with detectable β-lactamase activity^a^

|  | MOI 5 | MOI 25 |
| --- | --- | --- |
| BMMφ | 57 ± 4.6 | 67 ± 3.6 |
| BMDC | 53 ± 3.2 | 75 ± 4.4 |

a Cells were infected with *Y. pseudotuberculosis* secreting YopE-β-lactamase fusion protein. Detection of Yop secretion was done using membrane-permeable fluorescent β-lactamase substrate, CCF2-AM (Invitrogen).
